# Clinical Pathways for Oncological Gastrectomy: Are They a Suitable Instrument for Process Standardization to Improve Process and Outcome Quality for Patients Undergoing Gastrectomy? A Retrospective Cohort Study

**DOI:** 10.3390/cancers12020434

**Published:** 2020-02-13

**Authors:** Patrick Téoule, Emrullah Birgin, Christina Mertens, Matthias Schwarzbach, Stefan Post, Nuh N. Rahbari, Christoph Reißfelder, Ulrich Ronellenfitsch

**Affiliations:** 1 Department of Surgery, Universitätsmedizin Mannheim, Medical Faculty Mannheim, Heidelberg University, Theodor-Kutzer-Ufer 1-3, 68167 Mannheim, Germany; patrick.teoule@umm.de (P.T.); emrullah.birgin@umm.de (E.B.); st.post@icloud.com (S.P.); nuh.rahbari@umm.de (N.N.R.); christoph.reissfelder@umm.de (C.R.); 2 Department of General and Visceral Surgery, Städtisches Klinikum Karlsruhe, Moltkestr.90, 76133 Karlsruhe, Germany; christina.mertens@klinikum-karlsruhe.de; 3 Department of General, Visceral, Vascular, and Thoracic Surgery, Klinikum Frankfurt Höchst, Gotenstraße 6-8, 65929 Frankfurt, Germany; matthias.schwarzbach@klinikumfrankfurt.de; 4 Department of Visceral, Vascular and Endocrine Surgery, University Hospital Halle (Saale), Ernst-Grube-Str.40, 06120 Halle (Saale), Germany

**Keywords:** clinical pathways, gastric surgery, oncological gastrectomy, quality of care, outcomes, standardization

## Abstract

(1) *Background*: Oncological gastrectomy requires complex multidisciplinary management. Clinical pathways (CPs) can potentially facilitate this task, but evidence related to their use in managing oncological gastrectomy is limited. This study evaluated the effect of a CP for oncological gastrectomy on process and outcome quality. (2) *Methods*: Consecutive patients undergoing oncological gastrectomy before (*n* = 64) or after (*n* = 62) the introduction of a CP were evaluated. Assessed parameters included catheter and drain management, postoperative mobilization, resumption of diet and length of stay. Morbidity, mortality, reoperation and readmission rates were used as indicators of outcome quality. (3) *Results*: Enteral nutrition was initiated significantly earlier after CP implementation (5.0 vs. 7.0 days, *p* < 0.0001). Readmission was more frequent before CP implementation (7.8% vs. 0.0%, *p* = 0.05). Incentive spirometer usage increased following CP implementation (100% vs. 90.6%, *p* = 0.11). Mortality, morbidity and reoperation rates remained unchanged. (4) *Conclusions*: After implementation of an oncological gastrectomy CP, process quality improved, while indicators of outcome quality such as mortality and reoperation rates remained unchanged. CPs are a promising tool to standardize perioperative care for oncological gastrectomy.

## 1. Introduction

Gastric cancer is the fifth most common neoplasm and still ranks third among the world’s leading causes of cancer deaths, affecting approximately 783,000 people annually [1]. Regardless of improvements in surgical technique and perioperative management, surgery for gastric cancer remains challenging and patients who undergo radical resection are reported to have high complication rates [2,3]. One reason is that more and more elderly and multimorbid patients are resected [4,5]. On the other hand, due to preoperative malnutrition of patients with gastric neoplasms and chronic comorbidities, perioperative mortality can reach up to 8.8% [6]. Therefore, multidisciplinary perioperative management is required to reduce the risk of possibly severe perioperative complications during and after oncological gastrectomy. The implementation of clinical pathways (CPs) can potentially improve the quality of perioperative management [7]. CPs are specific instruments developed to improve the quality of outcomes of care by standardizing treatment processes. They can be defined as a protocol stipulating all tasks that should be carried out during a defined treatment [8,9,10]. The designated goal of CPs is to transfer evidence to the bedside. They comprise all disciplines involved in patient care [11,12]. For several gastrointestinal operations, CPs have proven advantageous with regard to perioperative outcomes [13]. Several studies have reported the results of patients undergoing oncological gastrectomy and treated with CPs. These studies showed a reduction in the length of stay (LOS) and reported a non-significant decrease in total complications, mortality and reoperation [14]. However, all of these studies were conducted in Asian countries. In Europe only a few studies have assessed the influence of multimodal management after gastrectomy. They were focused on laparoscopic gastrectomy or a comparative pre-CP group was missing [15,16,17,18].

Given that the expected effects of CPs must be considered specific to health systems, we performed a study in a German tertiary care hospital to evaluate an oncological gastrectomy CP with respect to its effects on process and outcome quality.

## 2. Results

### 2.1. Patient Characteristics

A total of 126 patients underwent oncological gastrectomy during the study period. The pre-CP group comprised 64 patients and the CP group involved 62 patients. Patient characteristics are displayed in Table 1. The clinical and demographic characteristics of both groups were comparable. The proportion of total gastrectomies was non-significantly higher in the pre-CP group, and correspondingly, there were proportionally more tumors extending to the entire stomach in this group. The type of surgical reconstruction differed significantly between the two groups. While all patients received a Roux-en-Y reconstruction, the proportion of handsewn esophagojejunostomies was higher in the pre-CP group (23.4%) than in the CP group (8.1%; *p* = 0.01).

### 2.2. Process Quality

Table 2 gives an overview of the comparison of the outcomes that reflect process quality. In the CP group, patients received liquid nutritional supplements significantly earlier (median 5.0 vs. 7.0 days in the pre-CP group; *p* < 0.0001). The usage of incentive spirometers increased following CP implementation, although the difference did not reach statistical significance (100% vs. 90.6% in the pre-CP group; *p* = 0.11). Foley and arterial catheters were removed significantly earlier in the pre-CP group (median of 1.0 vs. 4.0 and 2.0 vs. 5.0 days, respectively; *p* = 0.01).

### 2.3. Outcome Quality

Table 3 summarizes the results for outcome quality. There were two postoperative fatalities in the pre-CP group. Cause of death was respiratory failure following aspiration pneumonia in one case and multiorgan failure caused by sepsis following anastomotic leakage in the other. In the CP group, four patients died due to multiorgan failure caused by sepsis: one caused by duodenal stump leakage with severe peritonitis, one caused by aspiration pneumonia and myocardial infarction, one due to anastomotic leakage, and one due to bowel leakage with severe peritonitis.

Regarding outcome quality, groups differed significantly in three parameters. Median length of hospital stay (LOS) in the intermediate care and intensive care units was significantly shorter in the pre-CP group than the CP group (median stay 2.0 vs. 3.0, *p* = 0.0005; and 0.0 vs. 0.0, *p* = 0.01, respectively). The median of the highest measured visual-analogue-scale (VAS) pain score was significantly lower in the pre-CP group (5 compared to 6 in the CP group; *p* = 0.03). The readmission rate was higher in the pre-CP group (7.8% vs. 0; *p* = 0.05). No differences could be observed between groups with regard to postoperative morbidity and mortality. Additionally, groups did not differ regarding the summary measures for specific complications. The discharge goal of the CP could not be obtained and LOS did not differ between groups.

## 3. Discussion

This study assessed the effects of an oncological gastrectomy CP with regard to parameters of perioperative process and outcome quality. Because gastric surgery and the associated perioperative care are complex, it should only be done in a specialized setting by dedicated and experienced surgeons. A reduction in perioperative mortality has been observed in recent years. However, procedure-associated morbidity remains high and this is a relevant issue for patients and treatment teams [19,20]. The fact that much older and severely co-morbid patients, as well as patients in advanced tumor stages and with compromised performance status are resected might partly explain this fact [3,4,5]. Nevertheless, high morbidity and mortality might also be associated with insufficient standardization of perioperative treatment, and in particular with so called “failure to rescue”, a situation in which emerging complications are not detected and managed appropriately, resulting in the death of the patient [2,21,22,23,24]. Therefore, this study was designed to assess if implementing an oncological gastrectomy CP resulted in increased standardization of perioperative treatment and improved the process and outcome quality. Given that the relevant evidence is almost exclusively related to Asian countries [14,18,25], we conducted a study in a Germany tertiary care center.

In order to measure protocol adherence, process quality parameters were used as key performance indicators. Following CP implementation, we detected an improvement in some of these parameters, while others remained unaltered or even worsened.

A meta-analysis has shown that early enteral nutrition is associated with lower mortality and a shorter hospital stay after gastrectomy [26]. We observed a significantly earlier intake of liquid nutritional supplement, and a non-significantly earlier intake of soft and full diet after CP implementation. The incidence of postoperative pneumonia can be decreased by the use of incentive spirometers [27]. All patients used incentive spirometers after CP implementation, compared to only 90% in the pre-CP group. The fact that postoperative pneumonia did not decrease after CP implementation is therefore rather surprising. One potential explanation could be that more ASA III patients, who have a higher baseline risk for acquiring pneumonia, were operated on after CP implementation (56.1% vs. 46.7%). Given that ascending infections are related to indwelling catheters, early removal should be aimed for [28,29,30]. In our study, however, the median day when abdominal drains as well as peripheral and central venous catheters, epidural catheters and nasogastric tubes were removed remained unchanged after CP implementation. Drain fluid was checked for its amylase concentration on postoperative day 5 in all patients. Drains remained in situ in case of an elevated concentration. Therefore, a potential explanation for the delayed easy flow (EF) drain removal might be the higher proportion of pancreatic fistula in the CP group, with 11.3% vs. 6.3% for the pre-CP group, as well as duodenal stump leakage rate (3.2 vs. 0). In contrast to what was expected from CP implementation, two parameters showed an apparent decrease regarding their process quality. Foley and arterial catheters were removed on average one day later in the CP group. One hypothetical explanation for the delayed removal in patients treated with the CP could be that they stayed on average one day longer in intermediate care and intensive care units. A higher proportion of associated procedures and co-morbid patients could explain this fact.

Perioperative morbidity and mortality were not significantly different before and after CP implementation. While the 30-day mortality rate is frequently used, we employed the in-hospital mortality rate to account for prolonged treatment courses, which are common nowadays given advanced intensive care and interventional techniques. In-hospital mortality was 6.5% in the CP and 3.1% in the pre-CP group. This two-fold increase in mortality after CP implementation is worrisome. However, this observation is based on only two additional postoperative fatalities in the CP group, and the difference is not statistically significant. The result might therefore be spurious and must be interpreted with much caution. In comparison, the overall postoperative morbidity rate according to the Clavien-Dindo classification in our patients seems high. This can possibly be explained by the fact that this scheme counts every deviation from what is considered a normal postoperative course as a complication. Consequently, only 14 patients in the CP and 20 in the pre-CP group were classified as being without complications in our study.

The Enhanced Recovery After Surgery (ERAS^®^) society published perioperative care guidelines for gastrectomy [31]. These guidelines contain 25 care items compared to 23 items in our CP. Comparing the two documents, 17 recommendations are very similar, while six recommendations given by the ERAS guidelines are not included in our CP. Examples are as follows: surgical access type, transversus abdominis plane (TAP) block or the use of wound catheters, skin preparation, preanesthetic medication, prophylaxis for postoperative nausea and vomiting (PONV), and oral bowel preparation. In contrast to the ERAS guidelines, our CP comprises recommendations regarding vitamin B12 substitution, catheters, transfusion and nursing and rehabilitation. Possible future revisions of the CP should incorporate the evidence-based ERAS guidelines.

While the results regarding process quality were encouraging, three parameters related to outcome quality deteriorated after CP implementation. The LOS in the intermediate and intensive care units was significantly longer in the CP group. Moreover, the median of the highest visual-analogue-scale (VAS) pain score was significantly lower in the pre-CP group. This result is rather unexpected, given that the CP included a dedicated analgesia scheme according to recent recommendations. It also included epidural catheter placement, which was carried out in the overwhelming majority of patients. Additional oral analgesics were administered in a stepwise, pain-adjusted manner, so that there is no obvious explanation for higher pain levels in patients treated according to the CP. Therefore, a clear explanation for higher pain levels in the CP group is lacking. Hypothetically, nursing staff might have been more aware of possible postoperative pain after CP implementation, and consequently tended to carry out more accurate pain assessment, leading to a higher reported pain level. This would also explain why the stipulated goal of epidural catheter removal on day 3 was not met. This scenario could be regarded as ascertainment bias. On the other hand, extra requests for analgesics from patients did not differ between the groups treated with or without CP. This indicates that the stipulated analgesia scheme was quite sufficient. Inadequate pain management can lead to impaired mobilization, an increase in LOS, and ultimately, to elevated perioperative morbidity, particularly with regard to pulmonary complications.

CPs should also avoid excessively long LOS without medical reasons. In this study, we did not observe a decrease in LOS after CP implementation. However, a relevant variation in LOS was seen between individual patients. The stipulated goal for LOS in our CP might have been too ambitious, because it was clearly below the LOS reported in larger studies [14]. Moreover, the study comprised all consecutive patients, including those with severe postoperative complications. This may explain the large variation and exceedingly long LOS of some patients. The readmission rate was higher in the pre-CP group, which shows that patients treated with the CP were not discharged inappropriately early.

In summary, the implementation of a CP for oncological gastrectomy at our institution did not lead entirely to the results that were expected based on studies on gastrectomy CPs in Asian settings [32,33], and on studies on CPs for other procedures in abdominal surgery at our institution and in other settings [13,34,35,36,37,38,39,40]. The reasons for this apparent difference in the efficacy of gastrectomy and other abdominal surgery CPs can only be speculated on. It is known that the biology of the disease and care for patients undergoing gastrectomy for gastric cancer in Asia shows important differences compared to European settings [41], but it remains unclear which specific factors might have determined the lack of efficacy of our CP. Moreover, oncological gastrectomy potentially demands more complex perioperative care than other abdominal procedures, for which CPs have led to pronounced improvements in process and outcome quality [13,34,35,36,37,38,39,40,42,43]. From the results of this study, it is difficult to conclude if the lack of efficacy was mainly due to limited adherence to the CP, or due to its suboptimal content and design for the given setting.

One of the strengths of our study is that it included all consecutive patients undergoing oncological gastrectomy before and after CP implementation. This is comparable to the “intention to treat principle” in randomized trials. In the case where the individual goals of the CP were not met, the patient was not taken “off the pathway”. All patients who entered the study were analyzed regardless of deviations from the CP or possible complications. Therefore, selection bias is highly unlikely.

There are several methodological limitations inherent to the study. Its design is retrospective and included a single center. Moreover, we used chart review to collect data. This could compromise the validity of the data. Furthermore, the small sample size could bias the results. Documentation was not fully complete for all patients with regard to some variables and consequently, these could not be used for the analyses. Although selectively missing documentation is unlikely, bias could result. A crossover or, in other words, contamination bias could have occurred during the development and implementation phase of the CP. Health professionals who were part of the development team could have used their knowledge of the CP content prior to its implementation in October 2012. To counteract such issues, the CP was actually designed and implemented over only three months. Due to the study design with two groups of patients operated on before and after a defined time point, i.e., implementation of the CP, patients were operated on during different periods. The treatment during these periods might have been different (beyond the usage of the CP) because of other factors that influenced the process and outcome quality. For example, it is indisputable that surgical technique, and the skills as well as the experience of the individual surgeon have an effect on perioperative outcomes [44]. During the four-year study period, the surgeons who were in charge of and operated on patients changed. Therefore, surgical performance bias cannot be excluded.

Another weakness of our study is that not all stipulated goals were achieved after CP implementation. This suggests that not all team members adhered to the CP protocol. The main reasons for non-adherence to the main subitems have been explained above. Possibly, the addition of a dedicated study nurse to the CP team, and the introduction of an electronic CP could overcome non-adherence to the CP recommendations. The study nurse could promote protocol adherence and discuss the reasons for non-adherence with the appropriate caregivers. The use of an electronic CP checklist, with reminders in case of protocol deviation, could increase adherence, and thus potentially improve process and outcome quality.

Most of these limitations would have been avoidable if the study had been conducted as a randomized controlled trial. However, this is hardly feasible for studies evaluating CP usage in a single center because it usually requires cluster randomization [36,45].

## 4. Materials and Methods

### 4.1. CP Design, Implementation, and Content

Since 2006, the Department of Surgery, University Medical Center Mannheim, Medical Faculty Mannheim, Heidelberg University has implemented CPs for different surgical procedures in a stepwise manner [34,35,36,37,38,39,40,42,43,46,47]. In October 2012, a CP for oncological gastrectomy was introduced.

This CP is based on CPs for colorectal and bariatric surgery that incorporate ERAS elements. Both have been previously evaluated [36,43]. Specific elements were adapted to modify the CP for use in oncological gastrectomy. Both the original colorectal and the gastrectomy CP are based on published treatment and nursing recommendations. Furthermore, the best available evidence at the time of CP design was incorporated. The CP was designed and then implemented by a multi-hierarchical and interdisciplinary (anesthesiology, surgery, nutritional services, physiotherapy) team.

A literature review was done to identify current evidence on perioperative treatment elements. Subsequently, institutional standards that existed before, were integrated. Finally, all project participants agreed to the final CP version in a consensus meeting. Prior to the definitive implementation, all involved disciplines were trained to use the CP. After implementation of the CP, continuous efforts were made to enable further development and improvements of the CP based on suggestions made by staff.

A full version of the CP is provided in the online Supplementary Materials (Table S1). Its main contents are as follows: (1) hospital admission scheduled for the day before surgery; (2) epidural catheter placement; and (3) a stepwise oral pain medication scheme, based on non-opioids for all patients and on demand medication of potent opioids. Postoperatively, patients were transferred to a surgical intermediate care unit for at least one night. ICU admission took place only if deemed necessary by the surgeon and/or anesthesiologist. All patients were encouraged to drink sweetened tea until two hours prior to scheduled full anesthesia. An oral toluidine blue swallowing test was stipulated for postoperative day five. Drains were removed in case of a negative blue test and if respective enzyme levels in the drain fluid were not elevated (target drain: amylase <250 U/l in drain fluid). Detailed instructions on how to use an incentive spirometer were provided to patients. The stipulated day of discharge was postoperative day seven. Outpatient follow-up appointments were scheduled within 14 days after discharge. Patients were told to consult our emergency room in case of clinical irregularities. The rationale for incorporating the individual elements into the CP was that they were thought to either enhance recovery and thus shorten hospital stay, or to improve perioperative outcomes such as decreasing the risk of complications. Some of the elements (preoperative nutrition and smoking cessation, preoperative fasting and treatment with carbohydrates, epidural catheter placement, antithrombotic prophylaxis, antimicrobial prophylaxis, avoidance of hypothermia, glycemic control, urine catheter management, fluid balance, early and scheduled mobilization, and stimulation of bowel movement) are recommended in the consensus guidelines for enhanced recovery after gastrectomy of the Enhanced Recovery After Surgery (ERAS^®^) Group [31]. The perioperative analgesia scheme was endorsed by national guidelines. Other CP elements such as the oral toluidine blue swallowing test and abdominal drain management were based on pre-existing institutional standards, which were not backed by higher-level evidence. The targeted length of hospital stay was based on the minimum stay for oncological gastrectomy defined in the German DRG system [48].

The CP was designed as a four-page paper-based document containing all designated treatment steps for each pre- and postoperative day. CPs were kept with patients’ treatment charts, and therefore they were constantly available for all involved staff members.

### 4.2. Study Design

The study used a single-center retrospective cohort design. All consecutive patients undergoing elective oncological gastrectomy were included. The intervention group (CP group) comprised all patients operated on after CP implementation in October 2012 until September 2014. The control group (pre-CP group) included patients operated on before CP implementation (May 2010 to September 2012). No formal sample size calculation was done. Data were obtained by means of retrospective chart review.

Patients in the pre-CP group were treated according to the individual judgment and decisions taken by the treating surgeons. Several semiformal standards for selected elements of care (e.g., early removal of catheters, epidural analgesia and early mobilization) had been in place and were used prior to CP implementation, but there was no comprehensive tool covering the entire treatment continuum. In the CP group, all patients were treated according to the CP.

The study was approved by the ethical committee of the Medical Faculty of Mannheim of the University of Heidelberg (2015-823R-MA). Because of its retrospective nature, the requirement for informed consent to review medical records was waived by the ethical committee. Confidentiality of patient data was ensured. The study was conducted in compliance with the Declaration of Helsinki. Neither the individual de-identified participant data, nor the specific data are intended to be shared by the authors. The CP documents will be accessible indefinitely as online supplementary data. The study has been registered with the German Clinical Trials Register (DRKS00020323).

### 4.3. Patient Characteristics

Demographic and clinical characteristics included age, sex, and preoperative status of patients according to the American Society of Anesthesiologists (ASA) physical status classification [49], underlying disease, administration of neoadjuvant chemotherapy, tumor location, and serum albumin levels upon preoperative admission. Histopathological data were analyzed by the Department of Pathology, Universitätsmedizin Mannheim, Mannheim, Germany according to the 7th version of the TNM-classification [50].

### 4.4. Surgery

Both before and after CP implementation, surgery was carried out by dedicated upper GI surgeons with more than four years’ experience. To achieve R0-resection, patients received either total, distal or completion gastrectomy, depending on the anatomic location of the tumor and possible previous gastric operations. There were no laparoscopic resections. Associated procedures were performed when necessary. The gastrointestinal passage was preferably reconstructed using a long Roux-en-Y loop. Esophagojejunostomy was performed with a 25 mm circular stapler whereas gastrojejunostomy was hand sewn. A D2 lymphadenectomy according to the guidelines of the Japanese Gastric Cancer Association should be performed in all patients.

### 4.5. Study Outcomes

Process and outcome quality were defined according to the Donabedian model [51,52]. Process quality was considered as the adherence to treatment specifications as detailed in the CP and was assessed using the following parameters: day of removal of the foley catheter and epidural catheter, placement of central venous line and epidural catheter, postoperative mobilization, day of removal of intra-abdominal drainage and nasogastric tube, day of oral toluidine blue test, and day of resumption of liquid and solid diet.

Outcome quality was measured with the following parameters: morbidity, mortality, reoperation rate, LOS stratified by the presence or absence of complications, day of first postoperative defecation, pain level on a numeric rating scale and readmission rate. Morbidity was assessed according to the Clavien-Dindo classification of postoperative complications [53]. Deaths were counted as postoperative if they occurred during the hospital stay or during readmission. Surgical site infections were ascertained according to the Centers for Disease Control and prevention (CDC) definition [54]. Readmission was counted as such if it occurred no later than 30 days after initial discharge and if it was considered to be related to a postoperative problem.

### 4.6. Statistical Analysis

All outcomes were compared between the CP and pre-CP group. Missing values were not counted in the analyses with no imputation of missing values having been performed. Dichotomous variables were compared between groups using the chi-square test. Ordinal variables were compared using the Student’s *t*-test if they were normally distributed and the Mann-Whitney U-test if they were not normally distributed. For not normally distributed variables, the median was used for descriptive analyses. For normally distributed variables, the mean was used. *p*-values <0.05 were considered statistically significant. There was no adjustment for multiple testing. SAS 13.2 (Cary, NC, USA) was used for all statistical analyses.

## 5. Conclusions

This study showed that using a CP for oncological gastrectomy affects several aspects of perioperative treatment. A high degree of process standardization was achieved and the uptake of respiratory training and the timely initiation of enteral nutrition was ensured. Other expected changes such as better pain control, earlier mobilization and shorter LOS were not realized after CP implementation. Outcome quality measured with perioperative morbidity and mortality did not change after CP implementation. In conclusion, an oncological gastrectomy CP can be used to standardize perioperative care, but its utility must be carefully weighed against the anticipated cost and effort required for implementation and continuous development.

## Figures and Tables

**Table 1:** Characteristics of the study groups.

Patient Characteristic	Pre-CP Group (*n* = 64) %	CP Group (*n* = 62) %	*p*-Value
Age in years; median (range; IQR)	65.5 (30-85; 20)	65.0 (25-89; 21)	0.79
Sex			0.88
Male	40 (62.5)	38 (61.3)	
ASA score			0.16
I	6 (10.0)	1 (1.8)	
II	26 (43.3)	23 (40.3)	
III	28 (46.7)	32 (56.1)	
IV	0 (0)	1 (1.8)	
X	4	5	
Type of tumor			0.12
Adenocarcinoma	61 (95.3)	58 (93.5)	
Other	3 (4.7)	4 (6.5)	
Tumor location			0.05
Proximal part	21 (32.8)	26 (41.9)	
Middle part	1 (1.6)	3 (4.8)	
Distal part	13 (20.3)	9 (14.5)	
Entire stomach	28 (43.7)	19 (30.7)	
Remnant cancer	1 (1.6)	5 (8.1)	
Neoadjuvant chemotherapy	38 (59.4)	35 (56.5)	0.85
TNM classification for Adenocarcinoma	61 (95.3)	58 (93.5)	
Tumor stage			0.57
T0	3 (4.9)	5 (8.6)	
T1	11 (18.0)	14 (24.1)	
T2	9 (14.8)	10 (17.2)	
T3	27 (44.3)	21 (36.2)	
T4	11 (18.0)	8 (13.8)	
Nodal status			0.46
N0	25 (41.0)	32 (55.2)	
N1	11 (18.0)	8 (13.8)	
N2	13 (21.3)	8 (13.8)	
N3	12 (19.7)	10 (17.2)	
Metastasis			0.66
M0	53 (85.5)	52 (88.1)	
M1	9 (14.5)	7 (11.9)	
X	2	3	
Resectional status			0.74
R0	57 (93.4)	55 (91.7)	
R1	4 (6.6)	5 (8.3)	
X	3	2	
Type of resection			0.07
Total	40 (62.5)	26 (41.9)	
Subtotal	9 (14.0)	10 (15.1)	
Completion gastrectomy	1 (1.6)	5 (8.1)	
Trans-hiatally extended	14 (21.9)	21 (33.9)	
Type of lymphadenectomy			0.19
D2	40 (67.8)	28 (60.8)	
Partial D3	14 (23.7)	15 (32.6)	
Local	1 (1.7)	2 (4.3)	
None	4 (6.8)	0 (0)	0.07
X	5	17	
Associated procedure ^#^	6 (9.4)	11 (17.4)	0.16
Liver resection	2 (3.1)	2 (3.1)	1
Colon resection	2 (3.2)	5 (8.1)	0.36
Distal pancreatectomy and splenectomy	4 (6.3)	5 (8.1)	0.36
Reconstruction			
Roux-en-Y	64 (100)	62 (100)	1
Stapler	49 (76.6)	57 (92.9)	0.01 *
Handsewn	15 (23.4)	5 (8.1)	
Preoperative albumin mean (g/L)	35.1	35.9	0.4
(standard deviation)	(4.75)	(4.40)	
Mean number of resected lymph nodes	26.6	25.1	0.43
(standard deviation)	(10.11)	(10.70)	
Mean lymph node ratio (positive LN/ total LN)	18	12	0.43
(range)	(0–92)	(0–89)	

ASA = American Society of Anesthesiology; X = missing data; Pre-CP group = Pre-Clinical pathway group; CP group = Clinical pathway group; dignity others Pre-CP-Group = in declining order: two neuroendocrine tumors, one leiomyosarcoma; dignity others CP-Group = in declining order: two leiomyosarcomas, one leiomyoma, one gastric metastasis of kidney cell carcinoma; IQR = interquartile range; ^#^ = multiple answers are possible; g/l = gram/ liter; * = *p*-value ≤ 0.05.

**Table 2:** Parameters of process quality.

Patient Characteristic	Pre-CP Group (*n* = 64) %	CP Group (*n* = 62) %	*p*-Value
Usage of incentive spirometer	58 (90.6)	62 (100)	0.11
Median day of oral toluidine test	5	5	0.72
(range; IQR]	(5–6; 0.0)	(4.0–7.0; 0.0)	
Number of patients with positive oral toluidine test	1 (1.6)	0 (0)	1
X	10	8	
Median day of peripheral venous catheter removal	6.5	4	0.35
(range; IQR)	(1–44; 10)	(0–24; 4.5)	
Median day of PDA catheter removal	6	6	0.71
(range; IQR)	(1–10; 2)	(0–9; 3)	
Number of patients with PDA catheter	59 (92.2)	53 (85.5)	0.47
Median day of central venous catheter removal	7	7	0.57
(range; IQR)	(1–19; 3.0)	(1–33; 4.0)	
Number of patients with central venous catheter	61 (95.3)	58 (93.5)	0.71
Median day of arterial catheter removal	1	2	0.01*
(range; IQR)	(0–7; 1.0)	(1–8; 2.0)	
Number of patients with arterial catheter	55 (88.7)	59 (95.1)	0.11
Median day of foley catheter removal	4	5	0.01*
(range; IQR)	(1–11; 3.0)	(1–3; 3.0)	
Number of patients with foley catheter	61 (95.3)	57 (91.9)	0.7
Median day of nasogastric tube removal	1	1	0.42
(range; IQR)	(0–3; 0.0)	(0–3; 0.0)	
Number of patients with nasogastric tube	54 (84.4)	57 (91.9)	0.67
Median day of EF drain removal	7	7	0.81
(range; IQR)	(4–32; 2.0)	(5–55; 2.0)	
Number of patients with EF drain	49 (76.5)	55 (88.7)	0.07
Median day of first intake of liquid nutritional supplement	7	5	<0.0001 *
(range, IQR)	(2–14; 4.0)	(4–10; 2.0)	
Median day of first intake of soft diet	6	6	0.62
(range, IQR)	(2–15; 1.0)	(3–7; 1.0)	
Median day of first intake of full diet	9	8	0.34
(range, IQR)	(6–16; 3.0)	(6–46; 4.0)	
Median day of full mobilization	1	1	0.75
(range; IQR)	(0–2; 0.0)	(0–5; 0.0)	
X	1	1	

Pre-CP Group = Pre-Clinical pathway group; CP group = Clinical pathway group; PDA = peridural anesthesia; IQR = interquartile range; EF = easy flow; X = missing data; * = *p*-value ≤ 0.05.

**Table 3:** Parameters of outcome quality.

Patient Characteristic	Pre-CP-Group (*n* = 64) %	CP-Group (*n* = 62) %	*p*-Value
Readmission	5 (7.8)	0 (0.0)	0.05
Mortality	2 (3.1)	4 (6.5)	0.43
Postoperative morbidity according to the Clavien-Dindo classification			0.68
Grade 0	20 (31.3)	14 (22.6)	
Grade I	7 (10.9)	5 (8.1)	
Grade II	21 (32.8)	24 (38.7)	
Grade IIIA	10 (15.6)	10 (16.1)	
Grade IIIB	2 (3.1)	4 (6.5)	
Grade IVA	1 (1.6)	1 (1.6)	
Grade IVB	1 (1.6)	0	
Grade V	2 (3.1)	4 (6.5)	
Revisional surgery	2 (3.1)	6 (9.7)	0.16
Postoperative pneumonia	6 (9.4)	7 (11.3)	0.77
Postoperative pleural effusion	18 (28.1)	10 (16.1)	0.13
Postoperative wound infection	2 (3.1)	7 (11.3)	0.09
Anastomotic dehiscence (esophagojejunostomy)	2 (3.1)	3 (4.8)	0.67
Duodenal stump leakage	0 (0.0)	2 (3.2)	0.24
Postoperative pancreatic fistula	4 (6.3)	7 (11.3)	0.35
Patients received postoperative RBCC transfusion	15 (23.4)	13 (21.0)	0.83
Median number of postoperative transfused RBCC	0	0	0.7
(range, IQR)	(0–4; 0.0)	(0–6; 0.0)	
Median number of highest VAS-score of pain	5	6	0.03 *
(range)	(0–10; 3.0)	(0–10; 3.0)	
X	2	0	
Analgesics requested (mean number of supplemental requested doses during hospital stay)	0.24	0.31	0.31
(range)	(0–1.54)	(0–2.25)	
Median day of first defecation	4	3	0.92
(range, IQR)	(2–8; 1.0)	(1–7; 2.0)	
Median length of stay in IMC	2	3	0.0005 *
(range, IQR)	(1–26; 2.0)	(1–47; 4.0)	
Median length of stay in ICU	0	0	0.01 *
(range, IQR)	(0–29; 0.0)	(0–31; 0.0)	
Median length of stay	16	16	0.66
(range, IQR)	(8–55; 10.0)	(9–63; 11.0)	

Pre-CP-Group = pre-clinical pathway group; CP-Group = clinical pathway group; VAS = visual analogue scale; IMC = intermediate care unit; ICU = intensive care unit; RBCC = red blood cell concentrate; IQR = interquartile range; * = *p*-value ≤ 0.05.

## Supplementary Materials

The following are available online at https://www.mdpi.com/2072-6694/12/2/434/s1, Table S1: Clinical Pathway for oncological gastrectomy used in the CP group of the study.
