# Supplementary material for: Clinical Pathways for Oncological Gastrectomy: Are They a Suitable Instrument for Process Standardization to Improve Process and Outcome Quality for Patients Undergoing Gastrectomy? A Retrospective Cohort Study
Source: Cancers (Basel). 2020 Feb 13;12(2):434. doi: 10.3390/cancers12020434 (PMC7073178; doi:10.3390/cancers12020434)
Supplement: Supplementary file 1 [file cancers-12-00434-s001.pdf]

Supplementary Materials: Clinical Pathways for Oncological Gastrectomy: Are They a Suitable Instrument for Process Standardization to Improve Process and Outcome Quality for Patients Undergoing Gastrectomy? A Retrospective Cohort Study

Patrick Téoule, Emrullah Birgin, Christina Mertens, Matthias Schwarzbach, Stefan Post, Nuh N. Rahbari, Christoph Reißfelder and Ulrich Ronellenfitsch

Table S1. Clinical Pathway for oncological gastrectomy used in the CP group of the study.

Name:		Surname:		Date of Surgery:	Date of Discharge:	
**Gastrectomy**	**PRE-ADMISSION**	**INPATIENT TREATMENT admission**	**Day of surgery**	**1st-3rd postoperative day**	**4rd-7th. postoperative day**	**DAY OF DISCHARGE 7th postoperative day**	**POST-DISCHARGE**
DIAGNOSTICS/ **MONITORING**	□ medical history<br>□ clinical examination<br>□ laboratory (blood count, electrolytes, liver- and kidney-specific values, coagulation, HbA1c for all patients, CEA, CA 19-9, CA 72-4)<br>□ esophagogastroduodenoscopy with endosonography and sampling (histology, HER-2-status, helicobacter eradication if necessary)<br>□ CT-neck-chest-abdomen<br>□ MRI liver in case of suspicious/unclear liver lesions<br>□ chest X-ray, if suspicious-> chest CT<br>□ after staging: case review at multidisciplinary tumor conference<br>□ if albumin <30 g/l: presentation at nutrition clinic<br>□ consent for anesthesia (including epidural catheter), additional exams on demand	□ laboratory (blood count, electrolytes, liver- and kidney-specific values, coagulation; CEA, CA 19-9, CA 72-4 if not done pre-admission)<br>□ crossmatch blood and prepare 2 RCC<br>□ ECG if not already performed at pre-admission	*Intraoperative monitoring:*<br>□ BP/HR<br>□ Relaxation<br>□ Body temperature (aim >36°C)<br>□ BS (target 120-200 mg/dl) every hour, correct with infusion of 5%-glucose or insulin bolus<br>□ FiO_2 (desired value 0,7)<br>□ CVP<br>*Postoperative*<br>□ intermediate care unit<br>□ monitor BP/HR<br>□ monitor urinary excretion<br>□ monitor surgical drains/ epidural anesthesia<br>□ blood sugar measurements (target 150 mg/dl)<br>□ at night: small laboratory routine, blood count and coagulation<br>□ chest X-ray after central venous catheter insertion	*Daily:*<br>□ monitor BP/HR/ temperature three times a day.<br>□ check target drains<br>□ wound control (after POD 2)<br>*1st postoperative day*:<br>□ transfer to general ward, if possible<br>□ blood count, electrolytes, kidney-specific values, coagulation, CRP<br>*3st postoperative day*:<br>□ blood count, electrolytes, kidney-specific values,	□ monitor BP/HR/ temperature twice a day.<br>□ check target drains<br>□ wound control/ change dressings<br>*5th postoperative day*:<br>□ oral toluidine blue swallowing test<br>*6th postoperative day*:<br>□ blood count, electrolytes, kidney-specific values, coagulation, CRP	□ Monitoring RR/HF/Temp. morgens	□ medical history<br>□ clinical examination<br>□ temperature<br>□ if necessary adjuvant therapy<br>□ vitamin B12-depot substitution every 2-3 months I.M.<br>□ after splenectomy: vaccination with Pneumovax 23, Mencevax ACWY, Act-HiB

	□ define date of admission/surgery (patient management)			coagulation, CRP			
**ANAESTHESIA**			□ cefazolin 2g IV (in case of allergy ciprofloxacin 400 mg IV) / metronidazole 500 mg IV <u>30-60 minutes before surgery</u><br>□ prewarming<br>□ general anesthesia				
**LINES**			□ G16 venous cannula<br>□ central venous catheter<br>□ arterial cannulation<br>□ nasogastric tube<br>□ epidural anesthesia (Th 8-10)	*<u>1st postoperative day</u>*:<br>□ if applicable remove arterial cannula before transfer to general ward<br>□ removal of nasogastric tube<br>*<u>3st postoperative day</u>*:<br>□ remove thoracic epidural catheter (in the morning of day 3 after surgery)	*<u>6th postoperative day</u>*<br>□ remove central venous catheter		*<u>Family doctor (after POD 10)</u>*<br>□ removal of wound staples
**FOLEY CATHETER**			□ insertion of transurethral foley catheter before surgery	*<u>1st postoperative day</u>*:<br>□ remove transurethral foley catheter (in the morning of day 1 after surgery)			
**DRAINS**							

			□ 1 EF on esophagojejunostomy (facultative)		*5th postoperative day*<br>□ Removal of EF in case of negative blue test and amylase <250 U/l in drain fluid		□
**NUTRITION**	□ balanced diet<br>□ supplementary nutrition as required (nutrition clinic)	□ balanced diet<br>□ supplementary nutrition as required (nutrition clinic)	□ sweetened tea up to two hours prior to surgery<br>□ after surgery: fasting	*1st postoperative day*:<br>□ water/ tea in sips<br>*2nd+ 3rd postoperative day*:<br>□ drinking quantity >2000 ml	*4th postoperative day*<br>□ Shakes<br>□ drinking at will (quantity >2000 mL)<br>*5th postoperative day*<br>in case of negative blue test:<br>□ shakes<br>□ oatmeal<br>□ drinking quantity >2000 ml<br>*6th postoperative day*<br>□ mushy-gastrectomy-diet (rich in protein, low in fat, easily digestible carbohydrates)	□ balanced gastrectomy-diet (rich in protein, low in fat, easily digestible carbohydrates)	□ balanced gastrectomy-diet (rich in protein, low in fat, easily digestible carbohydrates)

					<u>*7th postoperative day*</u><br>□ balanced gastrectomy-diet (rich in protein, low in fat, easily digestible carbohydrates)		
**IV MANAGEMENT**			□ maintain normovolaemia during surgery<br>□ Intraoperative/ postanaesthesia care unit /IMC: glucose G5% IV if blood sugar <120 mg/dl, insulin perfusor, glucose G5% IV if blood sugar >160 mg/dl (according to endocrinological consultation)<br>□ Postoperative fluid management according to CVP (target <5 cmH$_2$0	□ according to fluid balance target: 2500ml in 24 hours (total volume)<br><br><u>*On 3th postoperative day*</u><br>□ parenteral supplemental nutrition (1500ml in 24 hours)	□ according to fluid balance<br>□ parenteral supplemental nutrition (1500ml in 24 hours) [only until 6 POD]	□ none	
**DEFECATION**				□ magnesium 5 mmol/l as solution po tid until first defecation<br>□ enema in case of unsuccessful defecation until POD 3	□		
**MEDICATION**	□ continue medication<br>□ stop coagulation inhibitors, in case of warfarin or direct oral anticoagulants:	□ home medication with stated restrictions	<u>*Before surgery:*</u><br>□ home medication up to two hours before surgery	<u>*1st + 2nd postoperative day*</u>:	□ home medication with stated restrictions	□ home medication<br>□ pantoprazole 40 mg p.o. 1-	□ home medication, restart anticoagulant

	Fraxiparine 0,1/ 10 kg body weight bid<br>□ continue Aspirin in case of corresponding medical history<br>□ Stop oral antidiabetic medication on admission day (Metformin 48h prior to surgery)<br>□ If splenectomy is planned: vaccination with Pneumovax 23, Mencevax ACWY, Act-HiB >14 days before surgery	□ Pantoprazole 40mg 1-0-0<br>□ if quick less 70 %, 10mg Konakion® per day for 3 days i.v.<br>□ BS (target 120-200 mg/dl) l<br>□ insulin scheme:<br>BS 180-210: +2 iE;<br>BS 210-240: +3 iE;<br>BS 240-270: +4 iE<br>BS 300-350: +6 iE<br>BS 350-400: +8 iE<br>basal insulin sc<br>□ in case of full anticoagulation and planned thoracic EDC: Fraxiparine 0,1 / 10 kg body weight only in the morning	(consider above mentioned exceptions)<br>□ premedication as recommended by anesthesiologist<br>□ Pantoprazole 40mg 1-0-0 i.v.<br>□ in the morning: no Fraxiparine	□ home medication with stated restrictions<br>□ if quick less 70 %, 10mg Konakion® per day for 3 days I.V.<br>□ BS (target 120-200 mg/dl)<br>□ insulin scheme:<br>BS 180-210: +2 iE;<br>BS 210-240: +3 iE;<br>BS 240-270: +4 iE<br>BS 300-350: +6 iE<br>BS 350-400: +8 iE<br>basal insulin sc<br>□ pantoprazole 40 mg p.o. 1-0-0 (in case of subtotal resection)<br>□ Fraxiparine 0,3 ml sc 0-0-1 (in case of full anticoagulation: Fraxiparine 0,1 / 10 kg body weight twice a day<br>□ in case of soft pancreas tissue or small duct (< 3 mm):	□ pantoprazole 40 mg p.o. 1-0-0 (in case of subtotal resection)<br>□ insulin scheme:<br>BS 180-210: +2 iE;<br>BS 210-240: +3 iE;<br>BS 240-270: +4 iE<br>BS 300-350: +6 iE<br>BS 350-400: +8 iE<br>basal insulin sc<br>□ Fraxiparine 0,3 ml sc 0-0-1 (in case of full anticoagulation: Fraxiparine 0,1 / 10 kg body weight twice a day; in case planned removal the next day no Fraxiparine at nighttime<br>□ pancreatic enzymes 3 x 25000 iE in case of steatorrhea	0-0 (in case of subtotal resection)<br>□ Fraxiparine 0,3 ml sc 0-0-1 (in case of full anticoagulatio n: Fraxiparine 0,1 / 10 kg body weight twice a day<br>□ pancreatic enzymes 3 x 25000 iE in case of steatorrhea	medication 14 days after surgery<br>□ pancreatic enzymes 3 x 25000 iE in case of steatorrhea<br>□ vitamin B12-depot substitution every 2-3 months I.M. (depending on drug level)

				Octreotide 3 x 100 µg sc<br>□ pancreatic enzymes 3 x 25000 iE in case of steatorrhea			
**TRANSFUSION**			□ if Hb <8 g/dl or cardiopulmonary disorders	□ if Hb <8 g/dl or cardiopulmonary disorders	□ if Hb < 8g/dl or cardiopulmonary disorders		
**ANALGESIA**							
**intravenous**			<u>*Intraoperative*</u><br>□ metamizole 1 g I.V.<br><br><u>*Postoperative*</u><br>□ metamizole 1 g I.V. 1-1-1-1<br>□ if needed on top: Perfalgan® 1 g I.V. 1-1-1-1<br>□ piritramide 7,5 mg IV only in case of failed epidural anesthesia	<u>*1st postoperative day*</u>:<br>□ metamizole 1 g I.V 1-1-1-1<br>□ if needed on top: Perfalgan® 1 g I.V. 1-1-1-1<br>□ piritramide 7,5 mg IV only in case of failed epidural anesthesia	□ to avoid		
**oral**				<u>*from 2nd postoperative day:*</u><br>***Pain ladder***<br><br>*Step 1:*<br>metamizole 4x1 g p.o, on demand. paracetamol 4x1g p.o.<br><br>*Step 2:*<br>additional oxycodone/naloxone 10/5 mg p.o., oxycodone	<u>*from 2nd postoperative day:*</u><br>***Pain ladder***<br><br>*Step 1:*<br>metamizole 4x1 g p.o, on demand. paracetamol 4x1g p.o.<br><br>*Step 2:*<br>additional oxycodone/naloxone 10/5 mg p.o., oxycodone	□ on demand metamizole p.o.	□ on demand metamizole p.o.

**EPIDURAL CATHETER**		5-10 mg on demand<br><br>*Step 3:* pain consultation<br><br>oxycodone/naloxone 5/2,5 mg at age>75	5-10 mg on demand<br><br>*Step 3:* pain consultation<br><br>oxycodone/naloxone 5/2,5 mg at age>75		
**REHAB**	□ thoracic epidural delivery pump: ropivacaine 0,2% + 20 µg sufentanil epidural (46 ml ropivacaine 0,2% + 4 ml sufentanil epidural = 0,4 µg sufentanil/ml), 3-7 ml/h	□ thoracic epidural catheter (T8-10) as stated before<br>□ remove thoracic epidural catheter in the morning of day 3 after surgery (in case of full anticoagulation pause Fraxiparine 24h before removal and 2-4h thereafter)			
			□ request rehabilitation treatment, when no adjuvant treatment is planned		□ inquire about rehabilitation treatment
**QUALITY MANAGEMENT/ MEDICAL REPORT/**	□ collect tissue samples for research<br>□ request pathology report (surgeon)<br>□ brief operation report (surgeon)		□ check pathology report<br>□ case review at multidisciplinar	□ hand discharge letter to patient<br>□ hand chart to chief	

**DRG (diagnosis related groups)**			□ detailed operation report (written by surgeon)		y tumor conference<br>□ prepare discharge letter for referring physicians (including postoperative recommendations and post discharge appointment)	resident for DRG coding	
**INFORMATION AND CONSENT**	□ avoid alcohol and smoking 14 days before surgery<br>□ informed consent<br>□ hand out patient brochure	□ pre-operation discussion	□ information of next-of-kin by surgeon (red sheet)<br>□ inform patient postoperatively<br>□ inform referring physicians			□ final discussion with patient and next-of-kin (histological result and postoperative recommendations)<br>□ phone call to referring physician	□ discuss histological result and further recommendations with patient (if not happened before), communicate further appointments
**NURSING patient admission/ discharge**		□ PAT-admission + information<br>□ PAT-history<br>□ „red sheet ": next-of-kin phone number	<u>*Postoperative:*</u><br>□ PAT-information<br>□ counseling/guidance<br>□ effectuation of orders from operative report		□ prepare discharge documents<br>□ schedule outpatient follow-up appointment on day 8 after surgery<br>□ discharge talk	□ patient discharge	
**medical round / elab-oration**		□ participation in ward round	□ participation in ward round	□ participation in ward round	□ participation in ward round	□ participation in ward round	

**documentation**		□ effectuation of ward round orders<br>□ print laboratory results	□ effectuation of ward round orders<br>□ print laboratory results	□ effectuation of ward round orders<br>□ print laboratory results	□ effectuation of ward round orders	□ effectuation of ward round orders<br>□ printout of laboratory results
**patient care**	□ insert CP sheet into inpatient chart	□ insert CP sheet into inpatient chart	□ document secondary diagnosis (DRG form)<br>□ document nursing activities	□ document secondary diagnosis (DRG form)<br>□ document nursing activities	□ document secondary diagnosis (DRG form)<br>□ document nursing activities	□ document secondary diagnosis (DRG form)<br>□ document nursing activities
**mobilization/ physiotherapy**		□ care according to nursing standard<br>□ drug administration	□ care according to nursing standard<br>□ drug administration	□ care according to nursing standard<br>□ drug administration<br>□ BT (spirometer prophylaxis, expectorant, Pine menthol)<br>□ thromboprophylaxis	□ care according to nursing standard<br>□ drug administration<br>□ BT (spirometer prophylaxis, expectorant, Pine menthol)<br>□ thromboprophylaxis<br>□ schedule discharge appointment at outpatient diabetes clinic	□ care according to nursing standard<br>□ drug administration<br>□ BT (spirometer prophylaxis, expectorant, Pine menthol)<br>□ thromboprophylaxis
**patient control**			□ mobilization: 5 h after surgery to edge of bed depending on age/GH/time	□ mobilization: >4h out of bed; walk on aisle twice,	□ complete mobilization (in bed only during nap and at night)	□ complete mobilization (in bed only during nap and at night)

**wound/drains**				depending on age/ GH<br>□ PT in case of COPD, impaired mobility, prolonged bedriddenness	□ PT in case of COPD, impaired mobility, prolonged bedriddenness		
		□ vital signs (HR, BP, temperature)<br>□ pain intensity (1-10)	□ vital signs (HR, BP, temperature)<br>□ pain intensity (1-10)	□ vital signs (HR, BP, temperature, breathing)<br>□ pain intensity (1-10)	□ vital signs (HR, BP, temperature)<br>□ pain intensity (1-10)	□ vital signs (HR, BP, temperature)<br>□ pain intensity (1-10)	□ vital signs (HR, BP, temperature)<br>□ pain intensity (1-10)
				□ change wound dressing	□ change wound dressing	□ change wound dressing<br>□ remove drain after order	□ remove wound staples after order

AC = anticoagulation, AP = alkaline phosphatase, BP = blood pressure, BT = breathing therapy, BS = blood sugar, ca. = circa, CRP = C-reactive protein, CVP = central venous pressure, DRG = diagnosis related groups, ECG = electrocardiogram, EDC = epidural catheter, EF = Easyflow-Drain, G = Gauge, GH = general health, HPB = hepato-pancreatico-biliary, HR = heart rate, I.V. = intravenous, I.M. = intramuscularly, PAT = patient, POD = postoperative day, PPPD = pylorus-preserving pancreatoduodenectomy, PT = physiotherapy, RCC = red cell concentrate, RT=respiratory therapy, sc = subcutaneous. This clinical pathway does not absolve therapists from their responsibility of impact, adverse effect, dosage, contraindications of substances for patients. Recommended dosage is for normal weight adults without contraindications.

© 2020 by the authors. Licensee MDPI, Basel, Switzerland. This article is an open access article distributed under the terms and conditions of the Creative Commons Attribution (CC BY) license (http://creativecommons.org/licenses/by/4.0/).
